# Crystallization and X-ray diffraction studies of a complete bacterial fatty-acid synthase type I

**DOI:** 10.1107/S2053230X15018336

**Published:** 2015-10-23

**Authors:** Mathias Enderle, Andrew McCarthy, Karthik Shivaji Paithankar, Martin Grininger

**Affiliations:** a Institute of Organic Chemistry and Chemical Biology, Buchmann Institute for Molecular Life Sciences, Goethe University Frankfurt, Max-von-Laue-Strasse 15, 60438 Frankfurt am Main, Germany; b Department of Membrane Biochemistry, Max-Planck-Institute of Biochemistry, Am Klopferspitz 18, 82152 Martinsried, Germany; c EMBL Grenoble, 71 Avenue des Martyrs, 38042 Grenoble CEDEX 9, France

**Keywords:** fatty-acid synthase, fatty-acid synthesis, multienzyme, tuberculosis, mycolic acid

## Abstract

While a deep understanding of the fungal and mammalian multi-enzyme type I fatty-acid synthases (FAS I) has been achieved in recent years, the bacterial FAS I family, which is narrowly distributed within the Actinomycetales genera *Mycobacterium*, *Corynebacterium* and *Nocardia*, is still poorly understood. This is of particular relevance for two reasons: (i) although homologous to fungal FAS I, cryo-electron microscopic studies have shown that bacterial FAS I has unique structural and functional properties, and (ii) *M. tuberculosis* FAS I is a drug target for the therapeutic treatment of tuberculosis (TB) and therefore is of extraordinary importance as a drug target. Crystals of FAS I from *C. efficiens*, a homologue of *M. tuberculosis* FAS I, were produced and diffracted X-rays to about 4.5 Å resolution.

## Abstract 2

Bacterial and fungal type I fatty-acid synthases (FAS I) are evolutionarily connected, as bacterial FAS I is considered to be the ancestor of fungal FAS I. In this work, the production, crystallization and X-ray diffraction data analysis of a bacterial FAS I are reported.

## 1. Introduction   

Fatty acids are important as principal components of cellular membranes, as post-translational modifiers of proteins, as a storage form of energy and as signalling molecules. The synthesis of fatty acids is performed in a catalytic cycle that is largely conserved in nature. Despite the conservation of the chemistry of synthesis, the structures of fatty-acid synthases (FAS) differ significantly in eukaryotes and bacteria. FAS are classified into type I and type II systems. Type I FAS are protein complexes of up to 2.7 MDa in size. The catalytic sites are embedded in an elaborate architecture and substrates are shuttled as covalently bound molecules to acyl carrier protein (ACP) domains. The other ‘conventional’ FAS system that is present in most bacteria and mitochondria is referred to as type II, in which separate monofunctional proteins perform the specific steps required for fatty-acid synthesis (Schweizer & Hofmann, 2004 ▸; Gago *et al.*, 2011 ▸).

FAS type I multi-enzyme complexes have been extensively analyzed in recent years by X-ray crystallographic and cryo-electron microscopic (cryo-EM) studies (Maier *et al.*, 2010 ▸; Grininger, 2014 ▸). Whereas the mammalian FAS I forms X-shaped homodimeric complexes, the microbial (CMN-bacterial and fungal) FAS I occurs in barrel-shaped multimeric complexes with *D*_3_ symmetry. Microbial FAS I is found in essentially two stoichiometries: an α_6_β_6_ heterododecameric complex occurring in most fungal FAS I and a homohexameric complex present in some fungi and in the CMN-group bacteria. The structure of the 2.6 MDa yeast FAS I has been determined by X-ray crystallography (Jenni *et al.*, 2007 ▸; Lomakin *et al.*, 2007 ▸; Johansson *et al.*, 2008 ▸, 2009 ▸; Leibundgut *et al.*, 2007 ▸). The 1.9 MDa bacterial FAS I structure was characterized by cryo-EM, and a structural model was obtained by docking a fungal FAS I homology model (Boehringer *et al.*, 2013 ▸). Cryo-EM studies of the conformational dynamics additionally contributed to the structural knowledge of FAS I systems (Brignole *et al.*, 2009 ▸; Gipson *et al.*, 2010 ▸; Ciccarelli *et al.*, 2013 ▸), particularly by showing that bacterial and mammalian FAS I are conformationally dynamic, which critically determines their molecular mode (Grininger, 2014 ▸).

**Figure 1:**
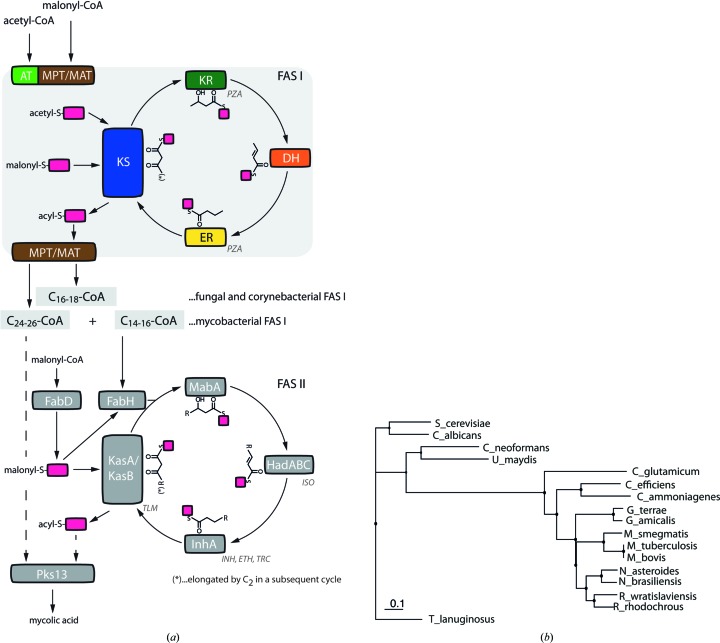
Overview of microbial *de novo* fatty-acid synthesis. (*a*) Synthetic machineries for fatty-acid production in bacteria and fungi. The grey background highlights the compartmentalized synthesis of acyl-CoA by the multifunctional FAS I systems. Key steps in the catalytic cycle of FAS I-mediated synthesis are as follows. The central ketoacyl synthase (KS) domain condenses ac(et)yl with malonyl to form β-ketobutyryl. Further, the compound is reduced by a ketoacyl reductase (KR) to β-hydroxyacyl, dehydrated by a dehydratase (DH) to produce enoyl, and again reduced by an enoyl reductase (ER). Substrate CoA esters are turned into ACP esters by transferases. ACPs (boxes in magenta) then shuttle substrates and intermediates to the active sites of the catalytic domains. Note that in FAS I, ACPs are part of the multienzyme. Also note that MPT (malonyl palmitoyl transferase) in fungal FAS I loads malonyl and unloads palmitoyl, and MAT (malonyl acyl transferase) in bacteria loads malonyl and unloads acyl chains of various chain lengths. The terminal thiol group of ACP is indicated to highlight that substrates and intermediates are provided as thioesters. Product specificities for corynebacterial, mycobacterial and fungal FAS I systems are given as reported in this study (*C. efficiens* FAS I) and previously: *C. ammoniagenes* FAS I (Stuible *et al.*, 1997 ▸), *M. tuberculosis* and *M. smegmatis* FAS I (Kikuchi *et al.*, 1992 ▸; Zimhony *et al.*, 2004 ▸; Peterson & Bloch, 1977 ▸) and *Saccharomyces cerevisiae* FAS I (Sumper *et al.*, 1969 ▸; Kresze *et al.*, 1977 ▸). Separate proteins of the FAS II are labelled as occurring in *M. tuberculosis* fatty-acid synthesis (Gago *et al.*, 2011 ▸; Bhatt *et al.*, 2007 ▸; Sacco *et al.*, 2007 ▸). Pharmaceutically relevant inhibitors of *M. tuberculosis* fatty-acid synthesis are included in the scheme: pyrazinamide (PZA; Sayahi *et al.*, 2011 ▸), isoxyl (ISO; Gannoun-Zaki *et al.*, 2013 ▸), thiolactomycin (TLM; Kremer *et al.*, 2000 ▸), isoniazid (INH), ethionamide (ETH) and triclosan (TRC) (Sullivan *et al.*, 2006 ▸; Banerjee *et al.*, 1994 ▸). (*b*) Phylogenetic analysis of selected bacterial and fungal FAS I. For the calculation of the phylogenetic tree, dual-chain fungal FAS I were submitted as β/α-fused pseudo-single chains. For the analysis, FAS I with the following GenBank accession Nos. were used: *Candida albicans* (X74952.1, L29063.1), *Saccharomyces cerevisiae* (CAA82025.1, CAA97948.1), *Cryptococcus neoformans* (AAW43793.1, AAW43793.1), *Ustilago maydis* (XP_759118.1), *Corynebacterium glutamicum* (YP_225128), *C. efficiens* (NP_739002.1), *C. ammoniagenes* (CAA46024.1), *Gordonia terrae* (WP_004019558.1), *G. amicalis* (WP_024498183.1), *Mycobacterium smegmatis* (AAO43178.1), *M. tuberculosis* (NP_217040), *M. bovis* (NP_856198.1), *Nocardia asteroides* (WP_019045581.1), *N. brasiliensis* (GAJ86521.1), *Rhodococcus wratislaviensis* (GAF47416.1) and *R. rhodochrous* (ETT26757.1). Analysis was performed using the neighbour-joining algorithm with the Jones–Taylor–Thornton substitution model using the *Ugene* software package (Okonechnikov *et al.*, 2012 ▸).

The bacterial FAS I system is only found in the CMN-group bacteria (Gago *et al.*, 2011 ▸; Figs. 1 ▸ *a* and 1 ▸ *b*). The most important representative of the FAS I-carrying bacteria is the highly pathogenic organism *Mycobacterium tuberculosis*, the causative agent of tuberculosis (TB). Functional studies have characterized *M. tuberculosis* FAS I as producing C_16_ and C_26_ fatty acids *de novo*, which serve as substrates for FAS type II-mediated and Pks13-mediated synthesis of the branched mycolic acids, which can be up to 90 C atoms in length (Kikuchi *et al.*, 1992 ▸; Bhatt *et al.*, 2007 ▸). The relevance of mycolic acids as an efficient barrier for *M. tuberculosis* is best illustrated by the use of three inhibitors of mycolic acid biosynthesis in antibiotic therapy for TB, pyrazinamide (PZA), isoniazid (INH) and ethambutol (Ma *et al.*, 2010 ▸), with PZA identified as specifically targeting *M. tuberculosis* FAS I (Sayahi *et al.*, 2011 ▸; Zimhony *et al.*, 2000 ▸; Fig. 1 ▸ *a*).

We recently reported the cryo-EM structure of *M. tuberculosis* FAS I (Ciccarelli *et al.*, 2013 ▸). For crystallographic studies, we identified *Corynebacterium efficiens* FAS I, which is 52% identical in sequence to *M. tuberculosis* FAS I, as a promising target. Here, we present the expression, purification, thermal stability data, crystallization and preliminary X-ray analysis of FAS I from *C. efficiens*.

## 2. Materials and methods   

### 2.1. Macromolecule production   

The cloning and purification of *M. tuberculosis* FAS I (UniProt accession code P95029) and *C. ammoniagenes* FAS I (UniProt accession code Q04846) have been described in detail elsewhere (Kikuchi *et al.*, 1992 ▸); *i.e.* cloning with an in-fusion cloning reaction (Takara Bio, Japan) and purification with engineered N-terminal Strep-II Tags (Schmidt & Skerra, 2007 ▸).

The coding sequence for *C. efficiens* FAS I (UniProt accession code Q8FMV7) was amplified by PCR from *C. efficiens* genomic DNA (DSM 44549). The gene was inserted into an SalI/XhoI-digested pME164 plasmid with an in-fusion cloning reaction (Takara Bio, Japan) to yield pME300 [pME164 is a pET-22b(+) (Merck, Germany) derivative with a N-terminal fused Strep-II Tag-encoding sequence]. Plasmid pME300 was modified using site-directed mutagenesis (forward primer GCG GCG CGC CGC AAC CAG CTG C; reverse primer GGT TGC GGC GCG CCG CGA CAC CCT CCA CGA GTG TC) to generate plasmid pME301 with S1778A and S1779A mutated ACP. Mutations were introduced to produce a highly homogeneous batch of non-post-translationally modified protein.

For the cloning of AcpS from *C. efficiens* in pET-coco-I, the coding sequence was prepared *via* PCR from genomic DNA (DSM 44549; forward primer AGA AGG AGA TAT AAG CAT GAT CTC GAT TGG AAC CGA TC; reverse primer TCG AGT GCG GCC TAG G CTA CCT GTT CTC GGT GGC CAC). The pET-coco-I plasmid was digested with SphI/AvrII and assembly was carried out with an in-fusion reaction to yield pME150.

Proteins were purified essentially as described elsewhere (Ciccarelli *et al.*, 2013 ▸). *Escherichia coli* plasmids pME300 and pME301 were transformed into BL21 Gold (DE3) cells (Agilent Technologies, USA). 35 ml LB medium precultures were inoculated with single ampicillin-resistant colonies and, after overnight growth, were used to further inoculate 2 l TB medium cultures. 2 l cultures were grown to an OD_600_ of 0.8–1.0 at 37°C and 180 rev min^−1^ and then cooled to 20°C and induced with a final concentration of 0.5 m*M* IPTG. After 16 h, the cells were harvested by centrifugation, frozen in liquid nitrogen and stored at −80°C. For purification, cells were resuspended in buffer *W* (0.1 *M* sodium phosphate pH 7.2, 0.15 *M* NaCl, 1 m*M* EDTA); protease inhibitor (Roche, Switzerland) and DNaseI (Applichem, Germany) were added before breaking the cells with a French press. Lysates were centrifuged for 1 h at 4°C at 45 000*g*. Supernatants were transferred onto a 10 ml affinity Strep-Tactin gravity-flow column (IBA, Germany), washed five times with one column volume of buffer *W* and eluted with 6 × 0.5 column volumes of buffer *E* (0.1 *M* sodium phosphate pH 7.2, 150 m*M* NaCl, 1 m*M* EDTA, 2.5 m*M* desthiobiotin). Proteins were polished by size-exclusion chromatography (SEC) using an ÄKTA­explorer (GE Healthcare, USA) on a Superose 6 XK 16/70 column (GE Healthcare, USA) with buffer *C* (0.1 *M* bis-tris propane pH 6.8, 0.2 *M* NaCl, 10% glycerol). Macromolecule-production information is summarized in Table 1 ▸.

For the preparation of enzymatically active protein, *C. efficiens* FAS I was co-expressed with *C. efficiens* AcpS protein. Expression was essentially performed as described above, except that both plasmids pME300 and pME150 were transformed and positive clones were selected by ampicillin/chloramphenicol double resistance.

**Figure 2:**
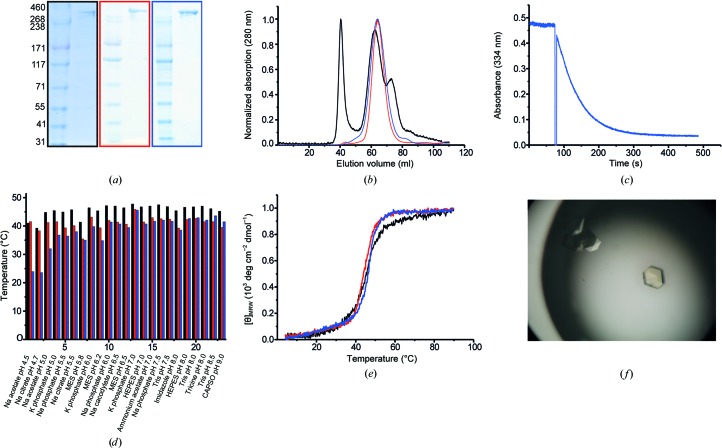
(*a*) Coomassie-stained SDS gel of purified proteins. Marker is shown in the left lanes (HiMark Protein marker; Life Technologies, USA; labelled in kDa). (*b*) Superposed size-exclusion chromatograms of proteins, normalized to 1 for the highest peak. The corynebacterial proteins run as monodisperse hexamers, while the *M. tuberculosis* FAS I chromatographic profile suggests conformational heterogeneity by additional aggregated and dimeric species. Slight shifts in elution volumes might result from different buffer conditions. Buffer conditions: *M. tuberculosis* FAS I, 0.1 *M* sodium phosphate pH 7.2, 0.1 *M* NaCl, 5 m*M* sodium malonate; *C. ammoniagenes*, 0.1 *M* sodium phosphate pH 6.5, 0.1 *M* NaCl, 5 m*M* sodium malonate; *C. efficiens*, 0.1 *M* bis-tris propane pH 6.8, 0.2 *M* NaCl, 10% glycerol. (*c*) Enzymatic activity of *C. efficiens* FAS I monitored by the consumption of NADPH at 334 nm. (*d*) Fluorescence-based thermal shift assay (Thermofluor assay) for buffer screening. (*e*) Thermal denaturation of protein monitored by CD spectroscopy. The melting points of the proteins were determined to be 45.4°C for *M. tuberculosis* FAS I, 44.6°C for *C. ammoniagenes* FAS I and 47.3°C for *C. efficiens* FAS I. (*f*) Crystals of *C. efficiens* FAS I. Colour code of figure: *M. tuberculosis* FAS I, black; *C. ammoniagenes* FAS I, red; *C. efficiens* FAS I, blue.

Protein stabilities were judged by circular-dichroism (CD) melting curves on a spectropolarimeter (J-715, Jasco, Japan) in buffer *S* (0.2 *M* sodium phosphate pH 7.2). Protein thermal denaturation by the Thermofluor method was carried out in a LightCycler 480 (Roche, Mannheim, Germany) and visualized using SYPRO Orange (Life Technologies, USA; Fig. 2 ▸ *d*; Ericsson *et al.*, 2006 ▸).

Preparations of *C. efficiens* FAS I from co-expression with AcpS were tested for FAS activity in a NADPH consumption assay (Lynen, 1969 ▸). For the assay, in a total volume of 100 µl, 25 µg FAS I in buffer *AB* (0.4 *M* potassium phosphate pH 7.3, 3 m*M* dithiothreitol) was incubated with 30 n*M* NADPH (Sigma–Aldrich, USA) and 50 n*M* acetyl-CoA (Sigma–Aldrich, USA). The absorption was recorded at 334 nm (Lambda 35, PerkinElmer, USA) for approximately 1 min before 60 n*M* malonyl-CoA (Sigma–Aldrich, USA) was added to the solution (Fig. 2 ▸ *c*).

For determination of the product spectra, reaction solutions with the above-described composition were incubated overnight at room temperature. After 16 h, C_17_-CoA (Sigma–Aldrich, USA) was added as an internal standard and the reactions were stopped by adding precooled (−20°C) acetone. These solutions were mixed for 20 s and stored for 60 min at −20°C. After centrifugation for 5 min at 20 000*g*, the supernatants were evaporated in a SpeedVac at 4°C. The CoA esters were dissolved in 60 µl Milli-Q water and, after ultrasonication, were analysed by HPLC-UV-MS [Ultimate 3000 RSLC (Thermo Fisher Scientific, USA) coupled to a micrOTOF-Q II system (Bruker Daltonics, Germany)]. CoA esters were separated on an RP-18 column (100 × 2.1 mm, particle size 1.7 µm; Waters, USA) in gradients of solvent *A* (water, 10 m*M* triethylamine/acetic acid buffer pH 9.0) and *B* (acetonitrile). Data were analyzed using the *DataAnalysis* 4.0 software package (Bruker Daltonics, Germany).

### 2.2. Crystallization   

Despite extensive crystallization trials, both *M. tuberculosis* and *C. ammoniagenes* FAS I gave crystals that diffracted to only about 8 Å resolution (data not shown). Hence, we chose another sequence homologue, FAS I from *C. efficiens*, for its reported moderate thermotolerance and the expected higher conformational stability of its proteins (Nishio *et al.*, 2003 ▸). Initial crystallization conditions for *C. efficiens* FAS I were identified by carrying out screening trials using various commercial sparse-matrix crystallization screening kits (The AmSO4 Suite, The Cations Suite, The JCSG+ Suite, The JCSG Core I–IV Suites, The PACT Suite, The PEGs Suite and The PEGs II Suite from Qiagen, Index HT from Hampton Research, and Morpheus HT-96 and The PGA Screen from Molecular Dimensions) using the sitting-drop vapour-diffusion method in 96-well Greiner plates at 4 and 22°C. Tiny crystals were observed in The PACT Suite at 4°C after 2 d. This condition was refined by the hanging-drop vapour-diffusion technique in 24-well plates using 1 µl protein solution in the droplet mixed with 1 µl reservoir solution. The best crystals were obtained at 0.1–0.3 *M* sodium malonate, 0.1 *M* bis-tris propane pH 7.5, 17–22% PEG 3350 at 4°C. Crystals grew after about one week with sizes ranging from 100 × 100 × 75 to 150 × 150 × 75 µm (Fig. 2 ▸ *f*). Crystallization information is summarized in Table 2 ▸. *C. efficiens* FAS I crystals were soaked with Ta_6_Br_12_ (Jena Bioscience), W_12_ (Alfa Aesar), W_18_ (a gift from Professor Robert Huber) or Nanogold (Nanoprobes) for 10 min, 1 h, 2 h or overnight. The concentrations of the soaking solutions for all clusters were varied in the low-micromolar range. For co-crystallization, the heavy atoms were added to the abovementioned crystallization conditions to a final concentration that was in the low-micromolar range. In both cases, the crystals showed poor diffraction (7 Å or worse) when exposed to X-rays and rapid decay in diffraction intensities after a few exposures. Data sets could not be collected.

### 2.3. Data collection and processing   

*C. efficiens* FAS I crystals were transferred to a cryo­protectant solution consisting of 20% ethylene glycol in the crystallization buffer, picked up in a nylon-fibre loop and plunged into liquid nitrogen. All crystals were exposed to single-wavelength X-radiation on beamline ID14-4 at the European Synchrotron Radiation Facility (ESRF) and maintained at 100 K while data were recorded on a CCD detector (ADSC Quantum Q315r). Individual diffraction images from *C. efficiens* FAS I crystals were initially indexed with *iMosflm* (Battye *et al.*, 2011 ▸) to determine the crystal form. Data were processed with the *xia*2 suite of programs (Winter, 2010 ▸) running the *XDS* package (Kabsch, 2010 ▸) and *AIMLESS* (Evans, 2006 ▸). Crystals gave two different crystal forms in space groups *R*32 and *C*2 with differing unit-cell parameters (Table 3 ▸), designated crystal forms I and II. All diffraction data are publicly available at Zenodo (http://dx.doi.org/10.5281/zenodo.20031).

## 3. Results and discussion   

We have recently established protocols for the recombinant overexpression of *M. tuberculosis* and *C. ammoniagenes* FAS I. *C. efficiens*, which is evolutionarily related to *C. ammoniagenes*, is reported to be moderately thermotolerant (Nishio *et al.*, 2003 ▸), and we expected a higher conformational stability of its protein inventory. Two FAS I-coding genes (FAS A, NP_737523.1, and FAS B, NP_739002.1) were identified in *C. efficiens* (Stuible *et al.*, 1997 ▸). FAS B (UniProt accession code Q8FMV7) was picked as a target for our studies owing to its higher sequence identity to *M. tuberculosis* FAS I (44 and 52% for FAS A and FAS B, respectively). About 5 mg of protein per litre of *E. coli* expression culture was obtained after chromatographic purification (Fig. 2 ▸ *a*). The hexameric conformation was monitored by size-exclusion chromatography (Fig. 2 ▸ *b*), and catalytic activity was demonstrated by spectroscopically monitoring NADPH consumption during the reductive steps in fatty-acid synthesis. The specific activity was determined as 270 ± 55 mU mg^−1^, which is comparable to previously reported values (Fig. 2 ▸ *c*; Stuible *et al.*, 1997 ▸). Under the assay conditions, *C. efficiens* FAS I produced C_16_-CoA (86%) and C_18_-CoA (14%). The protein stability of *C. efficiens* FAS I, as well as the homologous FAS I from *C. ammoniagenes* and *M. tuberculosis*, was analyzed by the fluorescence-based thermal shift (Thermofluor) assay and CD spectroscopy. Melting points of 45.4°C for *M. tuberculosis* FAS I, 44.6°C for *C. ammoniagenes* FAS I and 47.3°C for *C. efficiens* FAS I were determined, indicating low thermal stabilities of bacterial FAS I (Figs. 2 ▸ *d* and 2 ▸ *e*; Ericsson *et al.*, 2006 ▸). Aiming for the highest conformational homogeneity, the structure of *C. efficiens* FAS I was characterized on a mutant construct bearing the modifications S1178A and S1779A. These mutations prevent post-translational phosphopantetheinylation of the protein and should avoid the binding of intermediates or substrates to ACP that could induce different conformational states (Whicher *et al.*, 2014 ▸). This was carried out despite the observation that *C. efficiens* FAS I is inactive when expressed alone without *C. efficiens* AcpS.

Despite several optimization trials (in terms of protein purification, crystallization and post-crystallization protocols; *e.g.* the use of dehydration), diffraction data from the crystals could be acquired to only 4.5 Å resolution. Data sets from two crystals gave different unit-cell parameters in space groups *R*32 and *C*2, referred to as crystal forms I and II, respectively (Table 3 ▸). The packing of the FAS molecule in the asymmetric unit of the crystal was analyzed using *MATTHEWS_COEF* (Matthews, 1968 ▸) from the* CCP*4 suite (Winn *et al.*, 2011 ▸). For crystal form I, calculation of the Matthews coefficient (*V*_M_) gave values of 4, 2 and 1.3 Å^3^ Da^−1^, corresponding to solvent contents of 69, 38 and 7.1% for one, two and three molecules per asymmetric unit, respectively. This indicated the presence of one molecule in the asymmetric unit and the absence of noncrystallographic symmetry. In case of crystal form II, up to six chains (a whole hexamer barrel) can be accommodated in the asymmetric unit, indicating clear noncrystallographic symmetry. For crystal form II, search templates comprising the hexameric barrel, a trimeric dome, a dimer or a monomer individually did not yield unique solutions in the molecular-replacement analysis.

In the case of crystal form I, the phase problem could be solved by molecular replacement (MR) using the structural model of *M. smegmatis* FAS I (Boehringer *et al.*, 2013 ▸; PDB entry 3zen) as a model in *MOLREP* (Vagin & Teplyakov, 2010 ▸) with the help of the *CCP*4 suite. The MR solution was confirmed with *Phaser* (McCoy *et al.*, 2007 ▸), which gave an LLG score of 424 and a TFZ score of 26. It should be noted that the overall sequence identity between *C. efficiens* FAS I and *M. smegmatis* FAS I is 53% (with 67% positives and 4% gaps; data from *BLAST*; Altschul *et al.*, 1990 ▸). A pairwise alignment between the protein sequences of *M. smegmatis* FAS I and *C. efficiens* FAS I was used in *phenix.sculptor* (Bunkóczi & Read, 2011 ▸) from the *PHENIX* package (Adams *et al.*, 2010 ▸) to mutate the MR solution (PDB) file to the *C. efficiens* FAS I sequence. An immediate rigid-body refinement with the different domains (sequence ranges: AT, 10–367; ER, 368–879; SBS, 880–1002; DH, 1003–1289; MAT, 1290–1674; DM, 1953–2065; KR, 2073–2357; KS, 2358–3022) as independent rigid groups using *PHENIX* gave *R* and *R*_free_ values of 0.32 and 0.40, respectively. Although the diffraction data set is of low resolution, a preliminary view of the electron-density map displayed the different domains of this megadalton molecule. In the next step, we attempted model improvement using the *phenix.morph_model* command (Terwilliger *et al.*, 2012 ▸) invoking the *prime-and-switch* map (Terwilliger, 2004 ▸), but did not observe any significant improvement in either the refinement (*R*/*R*_free_) values or the electron density.

## Figures and Tables

**Table 1:** Macromolecule-production information

Source organism	*C. efficiens*
DNA source	Genomic DNA
Forward primer	CGA AAA AGG CGT CGA CGT GAC CGA ACC AGG CAG CAA CTT CGG G
Reverse primer	GGT GGT GGT GCT CGA GTT AGC CTT CGT AAC CGG TCG GCT TGA GG
Expression vector	pET-22b(+)
Expression host	*E. coli*
Complete amino-acid sequence of the construct produced	UniProtKB Q8FMV7

**Table 2:** Crystallization

Method	Vapour diffusion
Plate type	Greiner CrystalQuick plate (screening), Crystalgen SuperClear 24-well plates (final)
Temperature (K)	277 and 293
Protein concentration (mgml^1^)	10
Buffer composition of protein solution	0.1*M* bis-tris propane pH 6.8, 0.15*M* NaCl, 10% glycerol
Composition of reservoir solution	0.10.3*M* sodium malonate, 0.1*M* bis-tris propane pH 7.5, 1722% PEG 3350
Volume and ratio of drop	1:1 ratio protein:reservoir, 2l final volume
Volume of reservoir (l)	100 (screening), 600 (final)

**Table 3:** Data collection and processing Values in parentheses are for the outer shell.

	Crystal form 1	Crystal form 2
Diffraction source	ID14-4, ESRF	ID14-4, ESRF
Wavelength ()	0.939	0.939
Temperature (K)	100	100
Detector	ADSC Quantum Q315r	ADSC Quantum Q315r
Crystal-to-detector distance (mm)	628	680
Rotation range per image ()	1	0.2
Total rotation range ()	407	180
Exposure time per image (s)	1.2	0.3
Space group	*R*32	*C*2
*a*, *b*, *c* ()	337.9, 337.9, 246.7	243.9, 330.4, 214.1
, , ()	90, 90, 120	90, 115, 90
Mosaicity ()	0.3	0.1
Resolution range ()	506.0 (7.66.0)	204.5 (4.64.5)
Total No. of reflections	338393	339618
No. of unique reflections	13665	90165
Completeness (%)	100 (100)	100 (100)
Multiplicity	24 (25)	3.8 (3.8)
*I*/(*I*)	12 (1)†	5.2 (1.4)‡
*R*_r.i.m._	0.22 (4.2)	0.21 (1.1)
Overall *B* factor from Wilson plot (^2^)	350	162

† Data were scaled to a CC_1/2_ (Karplus Diederichs, 2012 ▸) of 0.5. The mean *I*/(*I*) falls below 2.0 at 6.5 resolution.

‡ Data were scaled to a CC_1/2_ (Karplus Diederichs, 2012 ▸) of 0.5. The mean *I*/(*I*) falls below 2.0 at 4.7 resolution.
